# Three Years with the Army of the Potomac—A Personal Military History

**Published:** 1912-10

**Authors:** William Warren Potter

**Affiliations:** Buffalo, N. Y., Brevet Lieutenant Colonel, U. S. Volunteers; Surgeon in Charge of First Division Field Hospital Second Army Corps ; Surgeon 57th Regiment New York Volunteers; Assistant Surgeon 49th Regiment New York Volunteers; Recorder Second Division Hospital Sixth Army Corps, etc., etc.


### Three Years with the Army of the Potomac—A Personal Military History.

By William Warren Potter, M.D., Buffalo, N. Y., Brevet Lieutenant Colonel, U. S. Volunteers; Surgeon in Charge of First Division Field Hospital Second Army Corps; Surgeon 57th Regiment New York Volunteers; Assistant Surgeon 49th Regiment New York Volunteers; Recorder Second Division Hospital Sixth Army Corps, etc., etc.

### *Deep Bottom, No.* 1.

We marched about three o'clock P. M. Tuesday, the 26th, crossing the Appomattox River at Point of Rocks at eleven o'clock at night, and the James before daylight at Jones' Neck, or Deep Bottom. The first Division had a fight early Wednesday morning, the 27th, capturing four twenty pounder Parrott guns, and a number of prisoners, losing about one hundred men killed and wounded. I rode in the leading ambulance Tuesday night, and was asleep when we crossed the James, the pontoons being muffled so the rumbling over the bridge did not even distrub me. I did not wake until the booming of the guns next morning suddenly aroused me, when I took a hasty cup of coffee and began work. It was expected that this movement would be such a surprise to the enemy that we could push on towards Richmond by this route, and possibly enter the city. But it appears the enemy had fortified a line across our path, and as it had become a part of reform policy on the part of Grant not to attack intrenched lines, we were foiled in the one object of the movement. But there was another which seemed to be prospering bettet. The mine that was constructing in front of Burnside's lines before Petersburg, was now completed and ready for firing. We, therefore, remained over the James River until more than one-half of the Confederate force was attracted hither in the belief that our attack in this direction was still real.

July 30. We remained at Deep Bottom until the night of the 29th, when, turning upon our heels, we marched rapidly back, reaching the lines in rear of the 9th Corps in time to witness the explosion of the mine this morning, which occurred soon after daylight. The bombardment and assault soon followed, and the 2d Corps was massed in reserve, expecting the order every moment to go into the support of the troops already swallowed up in the seething vortex of desctruction. When the mine was exploded, it gave forth a low, rumbling sound that shook the earth for a considerable distance, and sent up into the air a cloud of red sand and earth that resembled a water sprout at sea; and in this dusty cloud were men and guns, and material, mixed in inextricable confusion, all going to their death and destruction. The movement that the 2d Corps was momentarily expecting was suspended, owing to the failure of the Ninth Corps to do its work propertly, and we are back again at the Burchard House with our hospital, though it is expected that we may be compelled to move yet tonight. I have had no sleep to speak of for the past three nights, and am, therefore, a good deal worn down; so I hope we can rest for at least one night.

Monday, Aug 1. We packed up and started to move Saturday night but had gone only half a mile when the orders were countermanded, so we turned around and came back again. Last night orders were issued for the corps to be ready to move at a moment's notice, and it is the general impression that we shall move somewhere within the next twenty-four hours. Captain Wilson and Captain Elliot paid me a visit this morning, both in good spirits and handsome as ever. Colonel Beaver returned two days ago, but has not fully recovered from the effects of his wound and will, therefore, go back again in a day or two. The weather is very hot again, and we cannot be persuaded to do anything in the daytime that can be postponed until night, or some other good and convenient season.

Wednesday, August 3. The band came to play for the hospital today by order from Division Headquarters, and everybody enjoyed the music, notwithstanding the heat, including Colonel Beaver who is here. Mrs. General Barlow died in Washington last week, and General Barlow has gone North for fifteen days. Poor woman! she contracted her illness in the care of the soldiers; and I deeply sympathize with the General in his affliction. I have just learned of the death of Paymaster A. C. Winter, U. S. Navy, whose wife was Mollie Raynor, of Lansing, Michigan. Surely sad news comes in duplicate. When we were over the James at Deep Bottom the other day, I was within two miles of the iron-clad Cannonnicus, on board of which Dr. Adams is serving. I have drawn my pay for July, and shall send home $120.00 by Colonel Beaver who goes away tomorrow for twenty days, and will deposit it in the Express office in Washington for me.

Friday, August 5. Quietude reigns in the camp of the Army of the Potomac today. Indeed it is too hot for anything but quietude. I have invited a little dinner party this evening, among whom are Captain Favill, Captain Bronson and Captain Middleton, and shall serve a little extra menu, with ice cream and oranges for dessert. The dinner is given in honor of Favill, who goes out of service in a few days.

Monday, August 8. Dr. Dougherty expresses himself as anxious for me to return to service as an acting staff surgeon under the new order, and promises me the charge of the hospital if I will do so, but I have given him no encouragement in that direction. Yesterday Miss Hancock came up from City Point and spent the day at the hospital, and Miss Gilson called upon me last evening. Miss G. offers to come up and remain a week, caring for some of our bad cases, a kindness which will be duly appreciated. We can give her good quarters in the house, and, as she sings well and plays the piano beautifully, it will be quite a cheerful change to have her here, even for a short time. The failure at the mine explosion of July 30 is still the theme of conversation among the officers, but no one seems to know just where the responsibility rests; a Court of Inquiry will, I suppose, fix that in due time. A number of leaves of absence have been granted during the past month, among them one to Dr. Rowland, who goes home sick.

August 10. Wednesday. Everything is goiing on in the usual daily routine, though there was a little more firing than common on the lines last night and this morning. Yesterday a barge at City Point, loaded with ammunition, blew up by accident, killing and wounding about a hundred men. The explosion was terrific, and could be heard for miles away; we heard it distinctly, and are six or seven miles from the point. One surgeon going home on leave, and awaiting transportation, was blown to atoms. Captain Favill will be mustered out tomorrow, together with the greater part of his company. My groom, Corbleigh, will be mustered out on the 22d, but will remain until I go, to take care of my horses during transportation.

Friday, August 12. Yesterday I went to City Point to see Captain Favill off. Yes, Favill has gone! I am sorry, for he was a gentleman in the best sense of the word, and a companion who will be very much missed by all his friends. He has invited me to visit him in New York, at his father's, when my time expires. Dr. J. C. Norris, 81st Pa., my Assistant and Recorder, went home yesterday for twenty days. The day is intensely hot, as usual, not even a breath of air stirring. Lieutenant Alvord of General Caldwell's staff, whom we all remember with the kindest feelings, and who was married some time ago, is saddened by the grievous illness of his wife, who is reported in a dangerous condition. Lieutenant Hall was mustered out with Favill, and Captain Jones goes in a day or two; so the 57th is gradually melting away.

*Deep Bottom. No. 2.*

Monday, August 15. Here we are across the James again right where we were before, and we are called by the soldiers, "Hancock's Cavalry." The troops moved down to City Point on the afternoon of the 12th, took transports that night, and were sent up here and landed next morning, while the trains went overland and crossed on the bridges as before. We had a fight yesterday, and I have 160 wounded in my hospital. I amputated Captain James C. Bronson's right forearm yesterday, and he is now in my tent doing well. The day is intensely hot, almost to suffocation.

August 16. Today the cavalry and the First Brigade, General Miles, had a fight on the extreme right of our line near Charles City X Roads, the results of which I have not yet ascertained. I have about sixty additional wounded today, and the heat continues as great as ever. This afternoon a terrific thunderstorm burst over us, nearly collapsing the hospital tents. My own tent was in such extreme danger of going down, that I had four men hold it up by steadying the ropes at the corners, to keep it from crushing Bronson who is inside with an amputated arm. After the rain I witnessed an artillery duel between four or five of our gunboats, and some land batteries of the enemy. It occurred just at night, and was a splendid sight.

Saturday, August 20. Yesterday I was directed to superintend the shipment of the wounded of the Second Corps and the Cavalry Corps, which made a busy day of it. Two transports, the "Kent" and another, were moored at the wharf opposite my quarters that are on the bank of the river, and the wounded were carried on Board by the Ambulance Corps. The point where I am located is only two or three miles below Aiken's Landing, the latter being the place where I was delivered to the U. S. authorities, after being released by the rebels two years ago. After being in the front of Petersburg so long, where there is nothing but dust and fire—fire of the batteries—it is a delightful change to witness the transports and gunboats sailing up and down the river, at night blowing their whistles and displaying their parti-colored lights to avoid collision; it gives the scene a wonderfully commercial aspect, unlike the ordinary surroundings of war. One night I counted ninteen vessels from the top of the bank near my tent. Among the wounded sent away yesterday was Captain Bronson, who will never return to active service, though he could, and probably will, enter the veteran reserve corps. He gave me a fine new saddle cloth, breast strap, and other horse accoutrements. We have had rain for the last three days, and it continued all last night, so this morning the roads are not inviting to a long march.

Tuesday, August 23. We left Deep Bottom Saturday evening, marched all night, and next morning found ourselves at the Burchard House again; and, after a short halt for coffee, we marched to the extreme left, to support Warren's Fifth Corps in its struggle to take and hold the Weldon R. R. The rebels strove desperately to drive Warren off, assulting his positioin five times, and were as many times repulsed, with considerable slaughter. We have had much rain during the past week, which has served to make the roads very bad, but has not cooled the air; indeed, it is about as hot as ever. Dr. Gesner is back after his long absence, and Dr. Aiken has returned as an acting Staff Surgeon, taking charge of the Second Division hospital again.

